# Trends and Predictors of 90-Day Sepsis-Associated Readmissions Among Adults Hospitalized With Sepsis

**DOI:** 10.7759/cureus.115510

**Published:** 2026-08-31

**Authors:** Amin Farsani, Zin Htway, Alyssa Kozlowski, Arman A Sobhani

**Affiliations:** 1 Emergency Medicine, Los Robles Regional Medical Center, Thousand Oaks, USA; 2 Pathology, Los Robles Hospital, Thousand Oaks, USA; 3 Medicine, HCA Healthcare, Nashville, USA

**Keywords:** 90-day readmission, comorbidity index, hospital readmission, readmission after sepsis, sepsis

## Abstract

Background

Sepsis survivors remain vulnerable to recurrent hospitalization after discharge, yet factors associated with longer-term sepsis-associated readmission are incompletely understood. We evaluated temporal patterns and factors associated with 90-day sepsis-associated readmission following adult sepsis hospitalization.

Methods

We conducted a multicenter retrospective cohort study using de-identified electronic health record and administrative data from 50 hospitals between January 2018 and December 2025. Adults hospitalized with sepsis were eligible. Index hospitalizations in which the patient died during the hospitalization or was discharged to hospice were excluded from the readmission analysis. The primary outcome was recurrent hospitalization within 90 days in which sepsis, severe sepsis, or septic shock was coded as the primary diagnosis. Demographic, clinical, comorbidity, infection-related, and hospitalization-related variables were evaluated using multivariable logistic regression. Calendar year was initially modeled as a continuous predictor, with a sensitivity analysis treating year categorically to assess the assumption of a linear temporal relationship.

Results

Among 95,655 eligible index sepsis hospitalizations included in the readmission analysis, 6,095 (6.37%) were followed by a sepsis-associated readmission within 90 days. Index hospitalizations followed by readmission involved older patients, had greater comorbidity burden, longer index hospitalizations, and were more frequently discharged to non-home settings. Factors independently associated with increased readmission odds included greater Elixhauser Comorbidity Index, non-home discharge, pressure ulcer, severe sepsis, and abnormal lactate. Annual readmission rates demonstrated year-to-year variation, ranging from 5.52% in 2021 to 7.22% in 2023. When calendar year was modeled categorically, the overall year effect was significant (p < 0.001), with significantly higher adjusted odds of readmission in 2023 (OR 1.191, 95% CI 1.070-1.325) and 2024 (OR 1.136, 95% CI 1.019-1.266) compared with 2018. The categorical-year model provided significantly better fit than the continuous-year model (p < 0.001), indicating a nonuniform temporal pattern. In-hospital mortality among sepsis-associated readmissions was 32.3%.

Conclusions

Sepsis-associated 90-day readmission was associated with substantial mortality and multiple markers of post-sepsis vulnerability, including comorbidity burden, discharge disposition, and acute illness severity. These findings identify candidate factors associated with recurrent sepsis-related hospitalization and may inform future research on post-discharge risk assessment and intervention; however, prospective and external validation are required before these factors can be applied to individual patient-level risk prediction.

## Introduction

Sepsis is a life-threatening syndrome characterized by a dysregulated host response to infection, causing organ dysfunction, and remains a leading cause of hospitalization, mortality, and healthcare expenditure worldwide [1,2].

In the United States, sepsis affects more than 1.7 million adults annually and contributes to over 250,000 deaths [2]. Despite improvements in early recognition and management, including mandated sepsis care and evidence linking timely treatment to improved outcomes [3], survivorship after sepsis is increasingly recognized as a clinically distinct phase characterized by persistent physiologic vulnerability, functional decline, recurrent healthcare utilization, and excess long-term mortality [4-7]. This vulnerability may include phenomena described as post-hospital syndrome [8].

Hospital readmissions after sepsis represent a major component of this post-sepsis burden. Prior studies have reported substantial 30-day readmission rates, often ranging from 20% to 30%, with recurrent infection, exacerbation of chronic disease, and care-transition failures contributing to rehospitalization [6,9-11]. However, comparatively less attention has been directed toward 90-day readmission, which may better capture downstream consequences of chronic disease amplification and unresolved post-sepsis vulnerability.

Comorbidity burden may play a central role in this risk. The Elixhauser Comorbidity Index has been extensively validated for administrative datasets and captures multiple chronic disease domains associated with mortality, resource utilization, and readmission [12]. The Van Walraven score, a weighted derivative of the Elixhauser framework, provides an aggregate risk measure that may enhance discrimination by accounting for differential prognostic importance of individual comorbidities [13]. These measures may offer complementary insights into whether readmission risk is driven by accumulated chronic disease burden rather than acute illness severity alone.

At the same time, studies using diagnosis-based data face important interpretive limitations, particularly regarding attribution of infection source and causal relationships between index hospitalization and subsequent readmissions [14-16]. Evaluating readmissions in large multicenter cohorts while explicitly accounting for these limitations may help identify candidate markers of post-sepsis vulnerability and inform the development of future risk-assessment approaches while preserving appropriate interpretive caution.

Prior studies have suggested that both acute illness severity and baseline patient vulnerability contribute to post-sepsis outcomes. Greater organ dysfunction, septic shock, intensive care unit (ICU) admission, and elevated lactate levels have been associated with increased mortality and rehospitalization risk among sepsis survivors [4,6,9-11]. Similarly, discharge disposition has emerged as an important marker of post-discharge vulnerability, with patients discharged to skilled nursing facilities, rehabilitation facilities, and other non-home settings demonstrating higher rates of subsequent healthcare utilization and adverse outcomes [4,6,11]. Comorbidity burden, as measured by validated instruments such as the Elixhauser Comorbidity Index and Van Walraven score, has also been consistently associated with mortality, healthcare utilization, and readmission risk [12,13]. However, the relative contributions of acute illness severity, discharge disposition, and chronic disease burden to longer-term recurrent sepsis-related hospitalization remain incompletely characterized in large contemporary multicenter cohorts.

The present study specifically evaluated sepsis-associated rather than all-cause 90-day readmission, with the outcome restricted to subsequent hospitalizations in which sepsis, severe sepsis, or septic shock was coded as the primary diagnosis. We evaluated temporal patterns and factors associated with 90-day sepsis-associated readmission following adult sepsis hospitalization.

## Materials and methods

This was a multicenter retrospective cohort study utilizing de-identified electronic health record and administrative data from 50 hospitals between January 2018 and December 2025. The study was conducted in accordance with institutional policies for secondary analysis of de-identified data and was exempt from formal institutional review board review. This study is reported in accordance with the Strengthening the Reporting of Observational Studies in Epidemiology (STROBE) guidelines for observational studies.

Index sepsis hospitalizations were identified using administrative diagnosis codes for sepsis-related hospitalizations, including sepsis, severe sepsis, and septic shock. The study's diagnosis-based case definition was informed by contemporary Sepsis-3 definitions of sepsis and septic shock [1], while retaining administrative coding categories available in the dataset. Adult patients aged 18 years or older were eligible for inclusion. Index hospitalizations in which the patient died during hospitalization or was discharged to hospice were excluded from the primary readmission analysis because those hospitalizations were not at risk for subsequent readmission.

The primary outcome was sepsis-associated 90-day hospital readmission, defined as an inpatient hospitalization occurring within 90 days of discharge in which sepsis, severe sepsis, or septic shock was coded as the primary diagnosis. Readmission status was provided as a binary variable in the analytic dataset. Because the underlying data were derived from a cross-sectional billing dataset, the available de-identified analytic file did not permit us to independently verify whether readmissions were comprehensively captured across all 50 participating hospitals or whether individual patients could be longitudinally linked between participating institutions. Hospitalizations occurring outside the participating hospital network were also not known to be comprehensively observable. Accordingly, the analysis reflects sepsis-associated readmissions captured within the available dataset and may underestimate the absolute 90-day readmission rate.

The analytic dataset was structured at the hospitalization level, with each observation representing an eligible index sepsis hospitalization. Because the de-identified analytic dataset did not contain a persistent patient-level identifier, it was not possible to determine whether individual patients contributed more than one eligible index hospitalization during the study period. Hospitalization-level observations were therefore treated as independent in the regression analyses.

Demographic variables included age (continuous, years), sex (female versus male), race/ethnicity, and insurance status. Comorbidity burden was assessed using the Elixhauser Comorbidity Index [12], with a sensitivity analysis performed using the Van Walraven weighted comorbidity score [13]. Both comorbidity measures were modeled as continuous variables in their respective analyses.

Clinical measures available in the dataset included severe sepsis, septic shock, ICU admission, vasopressor use, and serum lactate status. Severe sepsis and lactate status were retained in the prespecified primary model as measures of acute illness severity. Septic shock, ICU admission, and vasopressor use were not additionally entered into the primary model because of their conceptual and clinical overlap with other measures of acute severity and treatment intensity. These variables are presented descriptively where available, and their unadjusted associations should not be interpreted as independent effects. Infection-related diagnosis flags included pneumonia, urinary tract infection, pyelonephritis, intra-abdominal infection, skin and soft tissue infection, and other documented infectious conditions.

Hospitalization characteristics included index hospital length of stay (continuous, days) and discharge disposition categorized as home versus non-home discharge. Calendar year of the index hospitalization was initially modeled as a continuous predictor to evaluate temporal patterns in sepsis-associated 90-day readmission. Because this specification assumes a linear relationship between calendar year and the log-odds of readmission, an additional sensitivity analysis was performed in which calendar year was modeled as a categorical variable, with 2018 serving as the reference year, while retaining the remaining covariates from the primary multivariable model. The fit of the categorical-year model was compared with that of the continuous-year model using a likelihood-ratio test to assess whether allowing year-specific effects significantly improved model fit.

Descriptive statistics were used to compare index hospitalizations with and without subsequent sepsis-associated 90-day readmission. Continuous variables were summarized as means and standard deviations and compared using independent-samples Welch t-tests. Categorical variables were summarized as frequencies and percentages and compared using Pearson's chi-square (χ²) tests. Corresponding test statistics and two-sided p-values were reported.

Multivariable binary logistic regression was performed to identify factors independently associated with sepsis-associated 90-day readmission. Variables included in the primary model were age, sex, race, insurance status, Elixhauser Comorbidity Index, severe sepsis, lactate status, pressure ulcer, pneumonia, urinary tract infection, pyelonephritis, index hospital length of stay, discharge disposition, and calendar year. These variables were selected a priori based on clinical relevance and previously reported associations with post-sepsis outcomes. Severe sepsis was retained as the principal diagnosis-based measure of acute illness severity; septic shock, ICU admission, and vasopressor use were not simultaneously entered because they represent overlapping measures of acute severity and treatment intensity. Continuous variables were modeled as continuous predictors, whereas categorical variables were entered as indicator variables.

Lactate status was categorized as normal, abnormal, or not available. Because lactate status was unavailable for a substantial proportion of the cohort and its absence may reflect clinical testing or data-availability patterns rather than random missingness, unavailable values were neither recoded as normal nor excluded from the primary analysis. Instead, lactate status was modeled as a categorical variable, with normal lactate serving as the reference category and separate indicator terms for abnormal and unavailable lactate status. This approach permitted retention of the full analytic cohort. Because lactate availability could vary over time, the distribution of lactate status (normal, abnormal, or not available) was additionally examined by calendar year. Differences in the distribution of lactate status across calendar years were assessed using Pearson's chi-square test. Hospital-specific patterns could not be evaluated because hospital identifiers were not available in the de-identified analytic dataset.

Adjusted odds ratios (ORs), 95% confidence intervals (CIs), Wald chi-square (χ²) statistics, and two-sided p-values were reported for the multivariable models. Model discrimination was evaluated using the area under the receiver operating characteristic curve (AUC), and model fit was summarized using McFadden's pseudo-R². Statistical significance was defined as a two-sided p-value < 0.05.

Because the Elixhauser Comorbidity Index and Van Walraven weighted comorbidity score are related measures of comorbidity burden, they were not included simultaneously in the same multivariable model. The Elixhauser Comorbidity Index was used in the primary model, and a sensitivity analysis was performed substituting the Van Walraven weighted comorbidity score [13] to assess the robustness of the findings.

An additional complete-case sensitivity analysis was performed among hospitalizations with available lactate status. Hospitalizations for which lactate status was not available were excluded from this analysis, resulting in an analytic cohort of 52,014 index hospitalizations. This analysis was performed to evaluate the robustness of the primary findings to the approach used for handling unavailable lactate data.

Hospital-level identifiers were not available in the de-identified analytic dataset. Consequently, hospital-specific effects and within-hospital correlation could not be explicitly modeled using clustered standard errors, generalized estimating equations, or mixed-effects methods. The primary logistic regression therefore treated hospitalization-level observations as independent. All statistical analyses were performed using IBM SPSS Statistics for Windows, version 29.0 (IBM Corp., Armonk, NY).

## Results

A total of 112,643 index sepsis hospitalizations were identified between 2018 and 2025. After excluding index hospitalizations in which the patient died during the hospitalization or was discharged to hospice, 95,655 index sepsis hospitalizations were included in the 90-day readmission analysis. Among these, 6,095 (6.37%) were followed by a sepsis-associated readmission within 90 days of discharge.

Index hospitalizations followed by sepsis-associated readmission involved patients who were older than those without readmission (64.36 ± 15.32 vs 59.72 ± 17.63 years; t = -22.647, p < 0.001), had a greater comorbidity burden as measured by the Elixhauser Comorbidity Index (4.79 ± 2.30 vs 3.58 ± 2.29; t = -39.802, p < 0.001), and had longer index hospitalizations (9.85 ± 9.78 vs 7.16 ± 8.18 days; t = -20.939, p < 0.001). Non-home discharge was also more common among index hospitalizations followed by readmission (67.8% vs 44.0%; χ² = 1303.835, p < 0.001) (Table 1).

**Table 1:** Baseline characteristics stratified by sepsis-associated 90-day readmission Continuous variables are presented as mean ± standard deviation and were compared using independent-samples Welch t-tests. Categorical variables are presented as n (%) and were compared using Pearson's chi-square (χ²) tests. Lactate percentages use the total number of index hospitalizations within each readmission group as the denominator. ICU = intensive care unit.

Characteristic	No 90-day readmission (n = 89,560)	90-day readmission (n = 6,095)	Test statistic	p-value
Age, years	59.72 ± 17.63	64.36 ± 15.32	t = −22.647	<0.001
Female	45,218 (50.5%)	2,915 (47.8%)	χ² = 16.188	<0.001
White race	54,505 (60.9%)	3,628 (59.5%)	χ² = 4.263	0.039
Hispanic ethnicity	9,443 (10.5%)	422 (6.9%)	χ² = 80.853	<0.001
Medicare/Medicaid	60,018 (67.0%)	5,062 (83.1%)	χ² = 674.915	<0.001
Private insurance	16,836 (18.8%)	629 (10.3%)	χ² = 274.870	<0.001
Elixhauser Comorbidity Index	3.58 ± 2.29	4.79 ± 2.30	t = −39.802	<0.001
Severe sepsis	32,736 (36.6%)	3,163 (51.9%)	χ² = 572.994	<0.001
Septic shock	10,285 (11.5%)	1,253 (20.6%)	χ² = 442.966	<0.001
ICU admission	20,900 (23.3%)	2,047 (33.6%)	χ² = 328.712	<0.001
Lactate status			χ² = 140.150	<0.001
Normal	29,280 (32.7%)	1,772 (29.1%)		
Abnormal	19,260 (21.5%)	1,702 (27.9%)		
Not available	41,020 (45.8%)	2,621 (43.0%)		
Length of stay, days	7.16 ± 8.18	9.85 ± 9.78	t = −20.939	<0.001
Non-home discharge	39,440 (44.0%)	4,135 (67.8%)	χ² = 1303.835	<0.001

Annual 90-day readmission rates demonstrated year-to-year variation during the study period (Figure 1), ranging from 5.52% in 2021 to 7.22% in 2023. Although calendar year was significantly associated with readmission when initially modeled as a continuous predictor (OR 1.021 per year, 95% CI 1.009-1.034; Wald χ² = 11.409, p < 0.001), sensitivity analysis treating calendar year as a categorical variable demonstrated a nonuniform temporal pattern.

**Figure 1:**
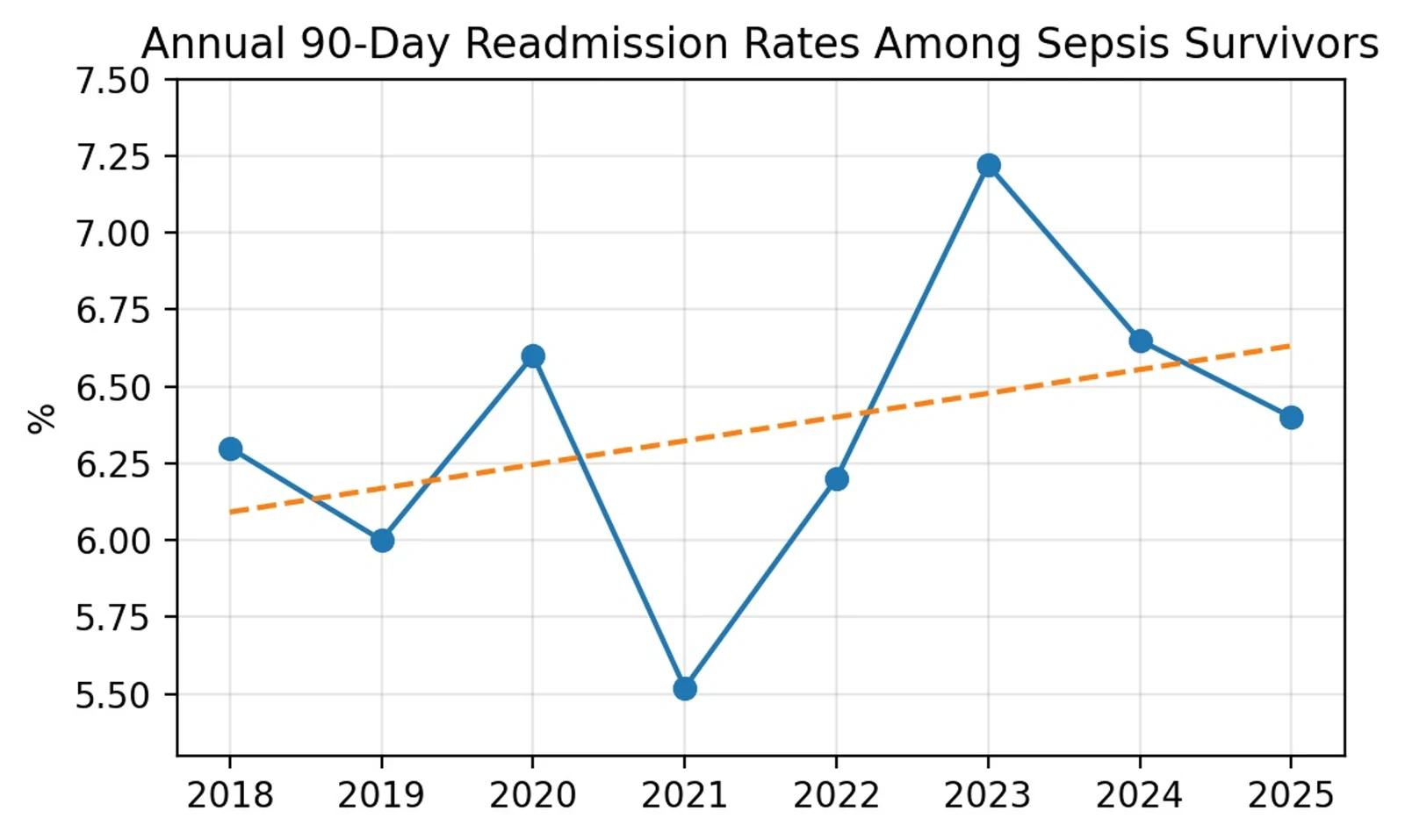
Annual 90-day sepsis-associated readmission rates following eligible index sepsis hospitalizations, 2018–2025 Annual readmission rates demonstrated year-to-year variation, ranging from 5.52% in 2021 to 7.22% in 2023. Sensitivity analysis treating calendar year categorically demonstrated a significant overall year effect and better model fit than the continuous-year specification, indicating that the temporal relationship was not adequately characterized by a uniform linear trend.

Using 2018 as the reference year, adjusted odds of readmission were significantly higher in 2023 (OR 1.191, 95% CI 1.070-1.325; p = 0.001) and 2024 (OR 1.136, 95% CI 1.019-1.266; p = 0.022), whereas differences for 2019, 2020, 2021, 2022, and 2025 were not statistically significant (Table 2). The overall categorical calendar-year effect was significant (Wald χ² [7] = 34.648, p < 0.001). Furthermore, the categorical-year model provided significantly better fit than the continuous-year model (likelihood-ratio χ² [6] = 23.035, p < 0.001), indicating that the temporal relationship was not adequately characterized by a simple linear trend.

**Table 2:** Sensitivity analysis of calendar year as a categorical predictor of sepsis-associated 90-day readmission Calendar year was modeled categorically, with 2018 serving as the reference year, while retaining the remaining covariates from the primary multivariable model. The overall categorical calendar-year effect was statistically significant (Wald χ² [7] = 34.648, p < 0.001). The categorical-year model provided significantly better fit than the continuous-year model (likelihood-ratio χ² [6] = 23.035, p < 0.001).

Calendar year	Adjusted OR	95% CI	Wald χ²	p-value
2018	Reference	—	—	—
2019	0.970	0.865–1.088	0.271	0.603
2020	1.076	0.963–1.204	1.676	0.196
2021	0.949	0.847–1.063	0.831	0.362
2022	0.985	0.880–1.103	0.068	0.795
2023	1.191	1.070–1.325	10.243	0.001
2024	1.136	1.019–1.266	5.256	0.022
2025	1.062	0.942–1.198	0.961	0.327

Multivariable logistic regression identified several factors independently associated with 90-day readmission. Greater comorbidity burden was associated with increased odds of readmission, with each one-point increase in the Elixhauser Comorbidity Index corresponding to a 12.9% increase in adjusted odds (OR 1.129, 95% CI 1.115-1.143; Wald χ² = 369.832, p < 0.001). Other prominent associations included non-home discharge (OR 1.731, 95% CI 1.623-1.846; Wald χ² = 279.444, p < 0.001), pressure ulcer (OR 1.937, 95% CI 1.779-2.109; Wald χ² = 231.766, p < 0.001), and severe sepsis during the index hospitalization (OR 1.447, 95% CI 1.369-1.529; Wald χ² = 170.508, p < 0.001) (Table 3).

**Table 3:** Multivariable logistic regression for sepsis-associated 90-day readmission Multivariable binary logistic regression was performed to identify factors independently associated with sepsis-associated 90-day readmission. Lactate status was modeled as a three-level categorical variable, with normal lactate serving as the reference category. Severe sepsis was retained as the principal diagnosis-based measure of acute illness severity; septic shock, ICU admission, and vasopressor use were not simultaneously included because they represent overlapping measures of acute severity and treatment intensity. The model included 95,655 index sepsis hospitalizations and 6,095 readmissions and demonstrated an AUC of 0.695 and McFadden pseudo-R² of 0.060.

Variable	Adjusted OR	95% CI	Wald χ²	P-value
Age (per year)	1.001	1.000–1.003	2.355	0.125
White race	0.895	0.847–0.946	15.549	<0.001
Female	0.914	0.867–0.964	10.820	0.001
Elixhauser Comorbidity Index (per point)	1.129	1.115–1.143	369.832	<0.001
Severe sepsis	1.447	1.369–1.529	170.508	<0.001
Lactate: abnormal vs normal	1.177	1.095–1.264	19.859	<0.001
Lactate: not available vs normal	1.122	1.053–1.195	12.553	<0.001
Pressure ulcer	1.937	1.779–2.109	231.766	<0.001
Pneumonia	1.160	1.093–1.230	24.135	<0.001
Urinary tract infection	1.226	1.155–1.300	45.310	<0.001
Pyelonephritis	0.717	0.544–0.945	5.573	0.018
Length of stay (per day)	1.002	0.999–1.005	1.722	0.189
Non-home discharge	1.731	1.623–1.846	279.444	<0.001
Private insurance	0.694	0.635–0.758	66.126	<0.001
Calendar year (per year)	1.021	1.009–1.034	11.409	<0.001

Lactate status was also independently associated with readmission. Compared with index hospitalizations with documented normal lactate status, those with abnormal lactate had higher adjusted odds of readmission (OR 1.177, 95% CI 1.095-1.264; Wald χ² = 19.859, p < 0.001). Index hospitalizations for which lactate status was not available also had higher adjusted odds relative to those with documented normal lactate status (OR 1.122, 95% CI 1.053-1.195; Wald χ² = 12.553, p < 0.001). Urinary tract infection (OR 1.226, 95% CI 1.155-1.300; Wald χ² = 45.310, p < 0.001) and pneumonia (OR 1.160, 95% CI 1.093-1.230; Wald χ² = 24.135, p < 0.001) were likewise associated with increased odds of readmission (Table 3). Although index hospital length of stay was greater among hospitalizations followed by readmission in the unadjusted comparison, it was not independently associated after multivariable adjustment (OR 1.002, 95% CI 0.999-1.005; Wald χ² = 1.722, p = 0.189).

Several factors were independently associated with lower odds of sepsis-associated readmission. Private insurance was associated with lower adjusted odds (OR 0.694, 95% CI 0.635-0.758; Wald χ² = 66.126, p < 0.001), as was pyelonephritis (OR 0.717, 95% CI 0.544-0.945; Wald χ² = 5.573, p = 0.018) (Table 3). These findings represent adjusted associations and should not be interpreted as evidence of causal effects.

Among sepsis-associated readmissions occurring within 90 days, in-hospital mortality was 32.3% (Table 4). Severe sepsis was present in 88.5% of readmissions, while septic shock occurred in 39.2%. The median time to readmission was 27 days (interquartile range, 10-52 days), indicating that a substantial proportion of recurrent sepsis-associated hospitalizations occurred relatively early after discharge.

**Table 4:** Outcomes of sepsis-associated readmissions within 90 days

Outcome	Value
In-hospital mortality	1,968 (32.3%)
Severe sepsis	5,395 (88.5%)
Septic shock	2,389 (39.2%)
Median time to readmission, days	27 (IQR 10–52)

The sensitivity analysis substituting the Van Walraven weighted comorbidity score for the Elixhauser Comorbidity Index yielded broadly similar findings (Table 5). Each one-point increase in the Van Walraven score was associated with increased odds of readmission (OR 1.038, 95% CI 1.035-1.041; Wald χ² = 525.897, p < 0.001). Associations with non-home discharge, pressure ulcer, severe sepsis, and abnormal lactate remained statistically significant. Calendar year also remained associated with readmission in this model (OR 1.016, 95% CI 1.003-1.028; Wald χ² = 6.186, p = 0.013).

**Table 5:** Sensitivity analysis using the Van Walraven weighted comorbidity score Multivariable binary logistic regression was repeated using the Van Walraven weighted comorbidity score in place of the Elixhauser Comorbidity Index. The model included 95,655 index sepsis hospitalizations and 6,095 readmissions and demonstrated an AUC of 0.702 and McFadden pseudo-R² of 0.063.

Variable	Adjusted OR	95% CI	Wald χ²	P-value
Age (per year)	1.001	0.999–1.003	1.799	0.180
White race	0.903	0.854–0.954	13.154	<0.001
Female	0.967	0.917–1.020	1.501	0.220
Van Walraven score (per point)	1.038	1.035–1.041	525.897	<0.001
Severe sepsis	1.434	1.357–1.516	161.957	<0.001
Lactate: abnormal vs normal	1.175	1.094–1.262	19.518	<0.001
Lactate: not available vs normal	1.106	1.038–1.178	9.635	0.002
Pressure ulcer	1.915	1.759–2.086	222.932	<0.001
Pneumonia	1.129	1.064–1.198	16.057	<0.001
Urinary tract infection	1.225	1.155–1.300	45.163	<0.001
Pyelonephritis	0.702	0.532–0.925	6.304	0.012
Length of stay (per day)	1.003	1.000–1.006	4.468	0.035
Non-home discharge	1.742	1.634–1.857	289.570	<0.001
Private insurance	0.698	0.639–0.763	63.786	<0.001
Calendar year (per year)	1.016	1.003–1.028	6.186	0.013

An additional complete-case sensitivity analysis restricted to index hospitalizations with available lactate status (N = 52,014) demonstrated broadly similar associations for major factors, including comorbidity burden, severe sepsis, non-home discharge, pressure ulcer, and abnormal lactate. However, the association between calendar year and 90-day readmission was attenuated and no longer statistically significant (OR 1.015, 95% CI 0.999-1.031; Wald χ² = 3.450, p = 0.063).

The distribution of lactate status differed significantly across calendar years (Pearson χ² [14] = 1064.680, p < 0.001) (Table 6). The proportion of index hospitalizations with unavailable lactate status increased from 32.9% in 2018 to 50.4% in 2025, with unavailable lactate status generally more frequent in the later study years. Hospital-level variation in lactate availability could not be assessed because facility identifiers were not available in the de-identified analytic dataset.

**Table 6:** Distribution of lactate status by calendar year among index sepsis hospitalizations included in the readmission analysis Lactate status was categorized as normal, abnormal, or not available. Percentages were calculated within each calendar year. The distribution of lactate status differed significantly across calendar years (Pearson χ²[14] = 1064.680, p < 0.001).

Calendar year	Total index hospitalizations	Normal lactate	Abnormal lactate	Lactate not available
2018	9,579	3,743 (39.1%)	2,689 (28.1%)	3,147 (32.9%)
2019	11,536	4,067 (35.3%)	2,901 (25.1%)	4,568 (39.6%)
2020	12,130	4,018 (33.1%)	2,608 (21.5%)	5,504 (45.4%)
2021	13,476	4,235 (31.4%)	2,561 (19.0%)	6,680 (49.6%)
2022	12,255	3,852 (31.4%)	2,501 (20.4%)	5,902 (48.2%)
2023	13,818	4,303 (31.1%)	2,889 (20.9%)	6,626 (48.0%)
2024	13,687	4,198 (30.7%)	2,897 (21.2%)	6,592 (48.2%)
2025	9,174	2,636 (28.7%)	1,916 (20.9%)	4,622 (50.4%)

The primary multivariable model included 95,655 index sepsis hospitalizations and demonstrated modest discrimination, with an AUC (area under the curve) of 0.695 and a McFadden pseudo-R² of 0.060. The model was developed to evaluate adjusted associations rather than for individual patient-level prediction and was not externally validated; therefore, these performance measures should not be interpreted as establishing a clinically validated risk-prediction tool. The sensitivity model substituting the Van Walraven weighted comorbidity score included the same 95,655 index hospitalizations and demonstrated an AUC of 0.702 and a McFadden pseudo-R² of 0.063.

## Discussion

In this large multicenter retrospective cohort study of sepsis hospitalizations across 50 hospitals between 2018 and 2025, 6.37% of eligible index sepsis hospitalizations were followed by a sepsis-associated readmission within 90 days, and these readmissions were associated with substantial in-hospital mortality. Several factors were independently associated with increased odds of readmission, including greater comorbidity burden, non-home discharge, severe sepsis, pressure ulcer, abnormal lactate status, and selected infection-related diagnoses. Private insurance and pyelonephritis were associated with lower adjusted odds. Collectively, these findings identify clinical and disposition-related characteristics associated with recurrent sepsis-related hospitalization and highlight post-discharge vulnerability following sepsis hospitalization.

Direct comparison of the observed 90-day readmission rate with previously reported all-cause sepsis readmission rates should be made cautiously. Unlike many prior studies evaluating all-cause rehospitalization, the present analysis focused specifically on readmissions in which sepsis, severe sepsis, or septic shock was coded as the primary diagnosis. This more restrictive outcome definition likely contributed to the lower observed readmission incidence. Accordingly, the findings may more specifically characterize recurrent sepsis-related hospitalization rather than overall post-sepsis healthcare utilization [6,9-11].

The findings are broadly consistent with previous studies demonstrating substantial post-discharge vulnerability and frequent rehospitalization among sepsis survivors [4,6,9-11], as well as with the broader critical illness survivorship literature [17]. Prior work has identified comorbidity burden, discharge disposition, and severity of illness as important correlates of readmission [9-11,17]. The present study extends these observations to a large contemporary multicenter cohort examining sepsis-associated readmission within 90 days of discharge.

Comorbidity burden was among the strongest factors associated with readmission. In the primary model, each one-point increase in the Elixhauser Comorbidity Index was associated with a 12.9% increase in adjusted odds of readmission. The sensitivity analysis substituting the Van Walraven weighted comorbidity score yielded a consistent association, supporting the relationship between underlying comorbidity burden and recurrent sepsis-related hospitalization. These findings suggest that vulnerability following sepsis may be influenced substantially by underlying chronic disease burden rather than by characteristics of the acute infectious episode alone [4,6,7].

Non-home discharge was also strongly associated with subsequent readmission. Index hospitalizations ending in non-home discharge may identify patients with greater functional impairment, residual illness, rehabilitation needs, or dependence on post-acute services. This finding is consistent with growing evidence that transitions of care are important determinants of outcomes among medically complex patients [4,6,11]. Although discharge disposition should not be interpreted as a causal determinant of readmission, it may serve as a readily identifiable marker of post-sepsis vulnerability.

The association between pressure ulcers and sepsis-associated readmission may reflect underlying patient vulnerability rather than a direct causal effect. In this context, pressure ulcer status may serve as a marker of greater clinical complexity, impaired functional reserve, or post-acute care needs that were not fully characterized in the available dataset. Accordingly, this finding should be interpreted as an association with clinical vulnerability rather than evidence that pressure ulcers independently cause subsequent sepsis-associated readmission.

Lactate status was independently associated with readmission, but lactate availability varied substantially over the study period. The proportion of index hospitalizations with unavailable lactate status increased from 32.9% in 2018 to 50.4% in 2025, and the distribution of lactate status differed significantly by calendar year. This temporal variation suggests that changes in lactate testing practices, documentation, or data capture may have contributed to the observed association between calendar year and readmission. This possibility is particularly relevant because the calendar-year association was attenuated and no longer statistically significant in the complete-case analysis restricted to hospitalizations with available lactate status. Hospital-level variation could not be assessed because facility identifiers were unavailable. Accordingly, temporal findings should be interpreted cautiously.

Several infection-related diagnosis flags were also associated with readmission after multivariable adjustment. Urinary tract infection and pneumonia were associated with increased odds, whereas pyelonephritis was associated with lower adjusted odds. These findings should not be interpreted as demonstrating causal effects of specific infection sources. Rather, these diagnosis flags may capture differences in patient characteristics, disease phenotype, coding practices, or other measured and unmeasured clinical factors. Further investigation would be required to determine the mechanisms underlying these associations.

Notably, index hospital length of stay was significantly longer among hospitalizations followed by readmission in the unadjusted analysis but was not independently associated after multivariable adjustment. This suggests that the unadjusted association may partly reflect differences in comorbidity burden, illness severity, discharge disposition, or other characteristics captured by the multivariable model rather than an independent effect of hospitalization duration itself.

Temporal patterns in sepsis-associated readmission were not uniform across the study period. Although modeling calendar year as a continuous predictor suggested an overall increase in adjusted readmission odds, a sensitivity analysis treating year categorically provided significantly better model fit and demonstrated that the association was concentrated primarily in 2023 and 2024 rather than reflecting a consistent year-over-year increase. The study period encompassed the COVID-19 pandemic and subsequent changes in healthcare utilization, sepsis epidemiology, hospital admission thresholds, resource availability, coding practices, and post-discharge care. In addition, lactate availability varied over time, and the continuous calendar-year association was attenuated in the complete-case lactate analysis. Accordingly, the temporal findings should be interpreted as evidence of year-to-year variation rather than a uniform secular increase in readmission risk.

The identified associations suggest that comorbidity burden, discharge disposition, and other indicators of clinical complexity may represent candidate markers of post-sepsis vulnerability. However, the present analysis was designed to identify associations rather than develop or validate a clinical risk-prediction tool, and these factors require prospective and external validation before being used for individual patient-level risk prediction. Future studies may evaluate whether candidate markers can support post-discharge interventions such as early outpatient follow-up, medication reconciliation, communication with post-acute facilities, standardized post-sepsis care plans, and surveillance for recurrent infection or clinical deterioration.

The high mortality observed during sepsis-associated readmission further emphasizes the clinical importance of these events. Nearly one-third of sepsis-associated readmissions ended in in-hospital death, while severe sepsis and septic shock were common. These findings suggest that recurrent sepsis-associated hospitalization represents clinically meaningful deterioration rather than healthcare utilization alone and support further investigation of strategies to improve transitions from inpatient to post-acute care.

The findings may also have implications for healthcare quality measurement. Current sepsis quality initiatives appropriately emphasize timely recognition and treatment during the index hospitalization. Given the frequency and adverse outcomes associated with recurrent sepsis-related hospitalization observed in this study, future research should determine whether sepsis-associated readmissions could serve as an informative quality measure and, importantly, what proportion of these events may be preventable through improved transitional care and survivorship programs. The present observational analysis cannot establish preventability or determine the extent to which individual hospitals should be accountable for subsequent readmissions.

This study has several strengths. The large multicenter cohort included data from 50 hospitals over an eight-year contemporary study period and permitted evaluation of temporal patterns and multiple demographic, clinical, and hospitalization-related factors. The analysis incorporated clinically relevant characteristics including comorbidity burden, illness severity, infection-related diagnoses, and discharge disposition. Robustness was further evaluated by substituting the Van Walraven weighted comorbidity score for the Elixhauser Comorbidity Index, performing a complete-case sensitivity analysis among hospitalizations with available lactate status, and evaluating calendar year categorically. Infection-related diagnosis flags were treated as covariates for adjustment rather than interpreted as causal determinants of readmission.

Several limitations should be considered. First, the retrospective observational design precludes causal inference. Although multivariable adjustment was performed, residual confounding from unmeasured factors such as socioeconomic status, functional status, frailty, outpatient follow-up, medication access, adherence, and other post-discharge characteristics may have influenced the observed associations.

Second, the underlying dataset was cross-sectional and based on hospital billing encounters rather than continuous longitudinal patient follow-up. The available analytic data did not permit confirmation that readmissions were comprehensively captured across all participating hospitals, and hospitalizations occurring at non-participating institutions may not have been captured. Consequently, some qualifying 90-day readmissions may have been missed, resulting in outcome misclassification and under-ascertainment of the absolute readmission rate. Accordingly, the observed 6.37% incidence should be interpreted as the rate of sepsis-associated readmissions captured within the available dataset rather than as a complete estimate of all sepsis-associated hospitalizations occurring within 90 days after discharge. The outcome definition also does not capture the full spectrum of all-cause post-sepsis healthcare utilization.

Third, the de-identified analytic dataset did not contain a persistent patient-level identifier; therefore, repeated eligible index hospitalizations contributed by the same individual could not be identified. Hospitalization-level observations were treated as independent. If some individuals contributed multiple eligible index hospitalizations, unaccounted-for within-patient correlation may have affected the estimated standard errors and statistical inference.

Fourth, although the study included data from 50 hospitals, hospital identifiers were not available in the de-identified analytic dataset. Therefore, we could not account explicitly for within-hospital clustering or evaluate institution-specific differences in readmission risk, coding practices, discharge patterns, patient case mix, sepsis management, or lactate availability. If outcomes and covariates were correlated within hospitals, failure to account for this clustering may have affected estimated standard errors and statistical inference. Future analyses using data with facility-level identifiers should evaluate the robustness of these associations using clustered or multilevel modeling approaches.

Fifth, lactate status was unavailable for a substantial proportion of index hospitalizations, and its availability varied by calendar year. The primary analysis retained unavailable lactate status as a separate category, avoiding classification of unavailable values as normal and preserving the full analytic cohort. Nevertheless, this approach does not eliminate the possibility of informative missingness. The complete-case sensitivity analysis yielded broadly similar associations for several major factors but attenuated the calendar-year association, underscoring the sensitivity of the temporal finding to the handling of unavailable lactate data.

Sixth, recurrent sepsis-related hospitalizations may represent new infectious episodes, progression of underlying chronic disease, healthcare-associated complications, or other intervening events occurring after discharge. The available data do not permit determination of the precise relationship between the index sepsis episode and the subsequent hospitalization. In addition, discharge to another facility could not be sufficiently subclassified to distinguish among different types of post-acute care and was therefore incorporated into discharge-disposition modeling rather than used as an exclusion criterion.

Seventh, the study period overlapped with the COVID-19 pandemic, which substantially altered healthcare utilization, hospital admission thresholds, sepsis epidemiology, resource availability, and post-discharge care. Temporal patterns may therefore have been influenced by pandemic-related factors that could not be fully accounted for.

Eighth, as with diagnosis-based retrospective analyses, coding variability and misclassification are possible. The direction and magnitude of any resulting bias cannot be determined from the available data.

Finally, the multivariable models were developed for association analysis rather than clinical prediction and were not externally validated. Although model discrimination was quantified using the AUC, these results should not be interpreted as establishing a validated risk-prediction tool. Prospective development and external validation would be required before the identified factors could be incorporated into individual patient-level risk prediction or clinical decision support.

Future studies should incorporate richer longitudinal data, patient- and hospital-level identifiers, clustered or multilevel analyses, and prospective validation to better distinguish potentially preventable readmissions from expected disease progression and to evaluate interventions targeting post-sepsis vulnerability. Given the strong association between non-home discharge and subsequent readmission, transitions of care for patients discharged to skilled nursing facilities, rehabilitation centers, and other post-acute settings may represent an important target for future quality-improvement initiatives. Potential strategies include dedicated sepsis nurse navigator programs, structured post-discharge follow-up, standardized post-sepsis care plans, reinforced return precautions, and improved communication between hospitals and receiving facilities. Prospective evaluation of these interventions will be necessary to determine whether they reduce recurrent sepsis-related hospitalization and improve post-discharge outcomes.

## Conclusions

Sepsis-associated 90-day readmission following an eligible index sepsis hospitalization was associated with substantial mortality and multiple markers of post-sepsis vulnerability, including comorbidity burden, discharge disposition, and acute illness severity. These findings identify candidate markers associated with recurrent sepsis-related readmission and may inform future research evaluating post-sepsis vulnerability and post-discharge interventions; however, prospective and external validation are required before these factors can be applied to individual patient-level risk prediction.
